# Integrative analysis of histopathological images and chromatin accessibility data for estrogen receptor-positive breast cancer

**DOI:** 10.1186/s12920-020-00828-4

**Published:** 2020-12-28

**Authors:** Siwen Xu, Zixiao Lu, Wei Shao, Christina Y. Yu, Jill L. Reiter, Qianjin Feng, Weixing Feng, Kun Huang, Yunlong Liu

**Affiliations:** 1 Institute of Intelligent System and Bioinformatics, College of Automation, Harbin Engineering University, Harbin, Heilongjiang China; 2 Center for Computational Biology and Bioinformatics, Indiana University School of Medicine, Indianapolis, IN USA; 3 Guangdong Provincial Key Laboratory of Medical Image Processing, School of Biomedical Engineering, Southern Medical University, Guangzhou, China; 4 Department of Medicine, Indiana University School of Medicine, Indianapolis, IN USA; 5 Department of Biomedical Informatics, The Ohio State University, Columbus, OH USA; 6 Department of Medical & Molecular Genetics, Indiana University School of Medicine, Indianapolis, IN USA; 7 Regenstrief Institute, Indianapolis, IN USA

**Keywords:** ATAC-seq, Chromatin accessibility data, Histopathological images, Integrative analysis, Computational biology, Bioinformatics

## Abstract

**Background:**

Existing studies have demonstrated that the integrative analysis of histopathological images and genomic data can be used to better understand the onset and progression of many diseases, as well as identify new diagnostic and prognostic biomarkers. However, since the development of pathological phenotypes are influenced by a variety of complex biological processes, complete understanding of the underlying gene regulatory mechanisms for the cell and tissue morphology is still a challenge. In this study, we explored the relationship between the chromatin accessibility changes and the epithelial tissue proportion in histopathological images of estrogen receptor (ER) positive breast cancer.

**Methods:**

An established whole slide image processing pipeline based on deep learning was used to perform global segmentation of epithelial and stromal tissues. We then used canonical correlation analysis to detect the epithelial tissue proportion-associated regulatory regions. By integrating ATAC-seq data with matched RNA-seq data, we found the potential target genes that associated with these regulatory regions. Then we used these genes to perform the following pathway and survival analysis.

**Results:**

Using canonical correlation analysis, we detected 436 potential regulatory regions that exhibited significant correlation between quantitative chromatin accessibility changes and the epithelial tissue proportion in tumors from 54 patients (FDR < 0.05). We then found that these 436 regulatory regions were associated with 74 potential target genes. After functional enrichment analysis, we observed that these potential target genes were enriched in cancer-associated pathways. We further demonstrated that using the gene expression signals and the epithelial tissue proportion extracted from this integration framework could stratify patient prognoses more accurately, outperforming predictions based on only omics or image features.

**Conclusion:**

This integrative analysis is a useful strategy for identifying potential regulatory regions in the human genome that are associated with tumor tissue quantification. This study will enable efficient prioritization of genomic regulatory regions identified by ATAC-seq data for further studies to validate their causal regulatory function. Ultimately, identifying epithelial tissue proportion-associated regulatory regions will further our understanding of the underlying molecular mechanisms of disease and inform the development of potential therapeutic targets.

## Background

Cancer heterogeneity results in tumors that exhibit distinct clinical features, therapeutic responses and patient outcomes. Understanding the factors involved in the onset and progression of cancers is pivotal for diagnosis and treatment. One of the main factors that contributes to the development of cancer is genetic changes [1–3]. However, the developmental process from genetic alterations to cancer phenotypes is complex and many mechanisms are still unknown. One way to uncover these mechanisms is through the integration of biomedical images with omics data [4–6].

Histopathology images are generally considered the gold standard for cancer diagnosis and grading in the clinic since they provide the distribution patterns of different tissues and cell types in the tumor microenvironment [7]. Previous studies have shown that spatial features, such as epithelial and stromal tissue proportion, derived from a single whole-slide tissue image represent rich histopathological information that can be quantified and used in statistical and biological analysis [8–10]. The identification and quantification of epithelial and stromal tissues on histopathological images can uncover spatial features of tumor phenotypes. These image-based features can be further integrated with genetic data to investigate the molecular regulatory mechanisms behind cancer phenotypes using statistical analysis methods.

The systematic integration of histopathological studies and omics profiles is expected to provide further understanding of tumor molecular biology and potentially more accurate stratification of patient prognoses. Recent reports have highlighted the significance of the contribution of stromal gene expression and morphological structure as powerful prognostic determinants for a number of tumor types [11–13]. However, gene expression signatures are affected by many factors, including the tumor environment, while gene regulatory landscapes are more stable among cells [14]. The regulatory landscape of a gene is specified by the overlying chromatin conformation, which may be more suitable for studying the potential effect of genomic changes at the bio-image level. To date, gene regulatory landscapes in tumors have largely been inferred through indirect means and little is known regarding the regulatory links between cancer gene expression and image features. During the past decade, the assay of chromatin accessibility has evolved into a powerful method to explore the regulatory landscape of primary human cancers [15–17]. The accessible genomic areas of chromatin are enriched with transcriptional regulatory elements which are crucial to gene expression, cell proliferation and tumor development. Several groups have reported that certain regulatory elements switch from inactive to active states (or vice versa) during the progression of diseases [18, 19]. This kind of global chromatin accessibility change can be detected and quantified by the assay for transposase-accessible chromatin using sequencing (ATAC-seq) [20]. Also, chromatin accessibility as a surrogate for regulatory element activity is arguably a continuous signal. In bulk sequencing, more reads aligning to a specific location of a chromosome would indicate more cells in the population have open chromatin at that particular site. We further inferred that such detectable chromatin accessibility differences, in turn, can induce changes in morphological features of tumor tissues that are quantified as spatial characteristics.

To study the association between chromatin accessibility changes and tumor phenotypes, images of tumor sections and ATAC-seq data from matched tumor samples are needed. The Cancer Genome Atlas (TCGA) contains histopathology images along with clinical outcomes and has recently generated high-quality ATAC-seq data in tumor samples from 54 estrogen-receptor (ER)-positive breast cancer (BRCA) patients. These large-scale experimental datasets make comprehensive integrative and correlative analyses feasible.

Most breast cancers are carcinomas that arise from the epithelial components of the lobules and ducts in mammary glands. Studies focused on developing tissue classification and segmentation algorithms have referred to tumors as epithelial tissues in image processing tasks [21, 22]. Following this terminology, we previously proposed a deep-learning-based image processing framework to estimate the epithelial tissue proportion on histopathological images for breast cancers. These image analysis results were used to analyze the relationship between epithelial tissue proportion and gene expression data [23]. Numerous genes were observed that were associated with the epithelial tissue proportion based on our pipeline. However, this analysis was not able to determine whether expression of these genes might be causal or might have resulted from changes in epithelial tissue proportion due to the complexity of the gene co-expression networks. To identify causal genes, additional analysis incorporating other omics information is required.

In this study, our aim was to identify key genomic regulatory regions that were associated with histological characteristics and thus, potentially impact clinical outcome. Such regions would be important for investigating the etiology of the associated disease and for identifying potential therapeutic targets.

In this work, we systematically explored the relationship between chromatin accessibility changes and epithelial tissue proportion. First, we used our new computational pipeline to quantify the epithelial tissue proportion from each sample. By performing correlation analysis, we observed that the change in chromatin accessibility of some specific open regions were strongly correlated with the change in epithelial tissue proportion across all samples. Then we implemented a strategy of linking DNA regulatory elements to their target genes based on the correlation of ATAC-seq and gene expression data. Downstream pathway analysis demonstrated that those target genes enriched in breast cancer-specific biological processes were associated with well-known oncogenes. Furthermore, we showed that the identified target genes could effectively predict overall survival of BRCA patients. In summary, the integration of the multi-omics data and histopathological images can provide new insights to explore the drivers and the molecular mechanism of ER-positive breast cancer.

## Results

### Overall strategy and image processing for the integrative analysis

**Fig. 1:**
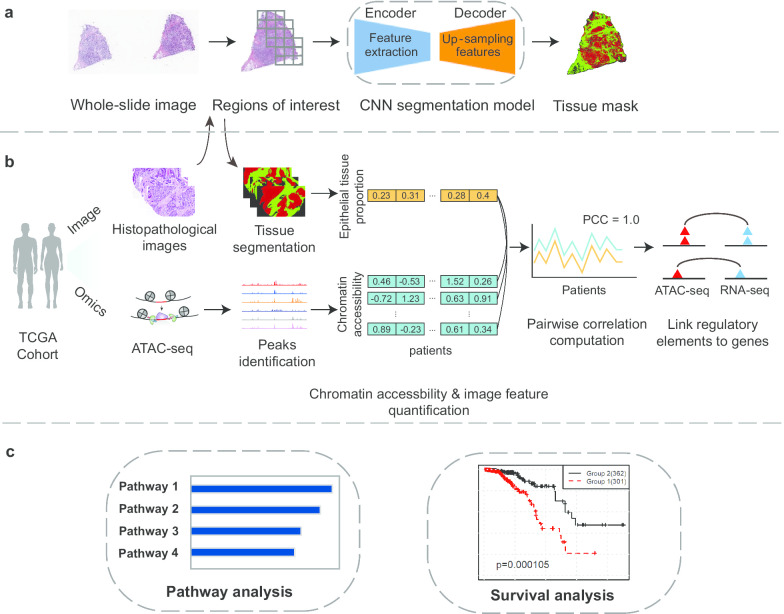
Study workflow. **a** Tissue segmentation and spatial feature extraction pipeline. For the tissue mask, red and green represent the epithelial and stromal tissue area, respectively. **b** Schematic diagram of the omics-image integration and correlation analysis. PCC = pairwise correlation coefficient. **c** Downstream pathway enrichment analysis and survival analysis using the target genes of the image feature-correlated DNA regulatory elements

The overall strategy of our integrative analysis comprises three stages as shown in Fig. 1. First, a convolutional neural network (CNN)-based model was used to identify the epithelial and stromal tissues from one whole-slide image for each patient. The epithelial tissue proportion was calculated based on the identified epithelial and stromal tissue area from the hemotoxin and eosin (H&E) stained slide. Second, to screen for the potential regulatory regions that share consistent correlation patterns with tumor development, we calculated the Spearman correlation coefficient between the epithelial tissue proportion and the quantitative chromatin accessibility for each detected open chromatin region in the ATAC-seq data across all 54 samples. Then, focusing on the significantly associated epithelial tissue proportion-open chromatin regions, we linked them to their potential target genes based on the Spearman correlation of ATAC-seq accessibility and gene expression values across all samples. Lastly, we conducted functional enrichment and pathway analysis to evaluate whether these potential target genes were enriched in BRCA-related pathways and well-known oncogenes. The target genes of epithelial tissue proportion-correlated regulatory regions were used to predict patient survival by performing a machine learning prognosis prediction method.

**Fig. 2:**
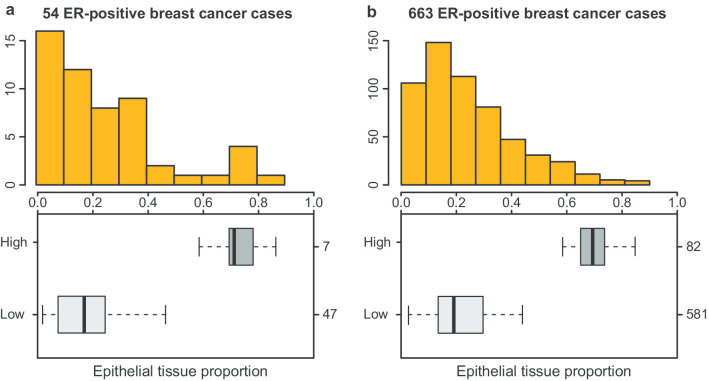
Distribution of epithelial tissue proportions for ER-positive breast cancer cases in TCGA. **a** Distribution of the 54 ER-positive breast cancer cases used in this study. **b** Distribution of all ER-positive breast cancer cases presently in TCGA. The histograms depict the distribution of epithelial tissue proportions, whereas the box plots stratify the cases into low and high epithelial groups using a cutoff *=* 0.5. Epithelial tissue proportions which were larger than or equal to 0.5 were classified as epithelial-high, while proportions smaller than 0.5 were classified as epithelial-low

We have previously developed an image processing pipeline that was used to classify and quantify epithelial tissue areas on histopathological images for all of the ER-positive breast cancer cases in TCGA. The image processing pipeline consists of three steps: 1) identification of a region of interest (ROI) on a whole-slide image; 2) patch-level segmentation of epithelial and stromal tissues in the ROI using a CNN model; 3) creation of a global tissue segmentation map by merging patch-level results followed by estimation of epithelial tissue proportions. For all 773 ER-positive patients, the previous image analysis results showed that these cases were enriched with stromal tissues, with a mean epithelial tissue proportion lower than 0.3 [23]. Here, we specifically focused on the epithelial tissue proportion of the ER-positive cases with paired ATAC-seq and image data (*n* = 54) to identify the associated open chromatin regions. The epithelial tissue proportion data of the 54 TCGA ER-positive breast cancer cases is provided in Additional file 2: Table S1. The distribution of epithelial tissue proportions for the 54 ER-positive breast cancer cases used in this study compared to all 773 cases in TCGA is shown in Fig. 2. Epithelial tissue proportions which were larger than or equal to 0.5 were classified as epithelial-high, while proportions smaller than 0.5 were classified as epithelial-low (Additional file 1: Fig. S1). We observed that 87% (47/54) of the cases used in this study had low epithelial tissue proportions (values smaller than 0.5) compared to 89% (691/773) of all ER-positive cases in TCGA. Therefore, the distribution of epithelial tissues in the 54 cases used in this study appears to be representative of the entire TCGA ER-positive group.

### Correlation analysis reveals the open chromatin regions related to epithelial spatial characteristics

For each detected open chromatin region, we asked whether this potential regulatory region might have contributed to tumor development in ER-positive breast cancer patients. To begin to address this question, we implemented canonical correlation analysis between the quantitative chromatin accessibility measure and the epithelial tissue proportion across all 54 patients.

The Spearman correlation coefficient, r, was used to evaluate whether the potential regulatory regions were significantly correlated with the epithelial tissue proportion. A total of 215,920 open chromatin regions were analyzed. Multiple testing correction (FDR < 0.05) is used to account for false positives. This analysis showed that 436 regulatory regions were significantly correlated with the epithelial tissue proportion, 111 of which were positively correlated and the other 325 were negatively correlated. The ATAC-seq peak signal data of 54 TCGA ER-positive BRCA cases is provided in Additional file 2: Table S2 and a complete list of these regulatory regions can be found in Additional file 2: Table S3. In addition, the peak ID, the start and end positions of the peak, and the *p*-value and the FDR of the correlation analysis, can be viewed on our RShiny website (https://yunlongliulab.shinyapps.io/omics-image/).

**Fig. 3:**
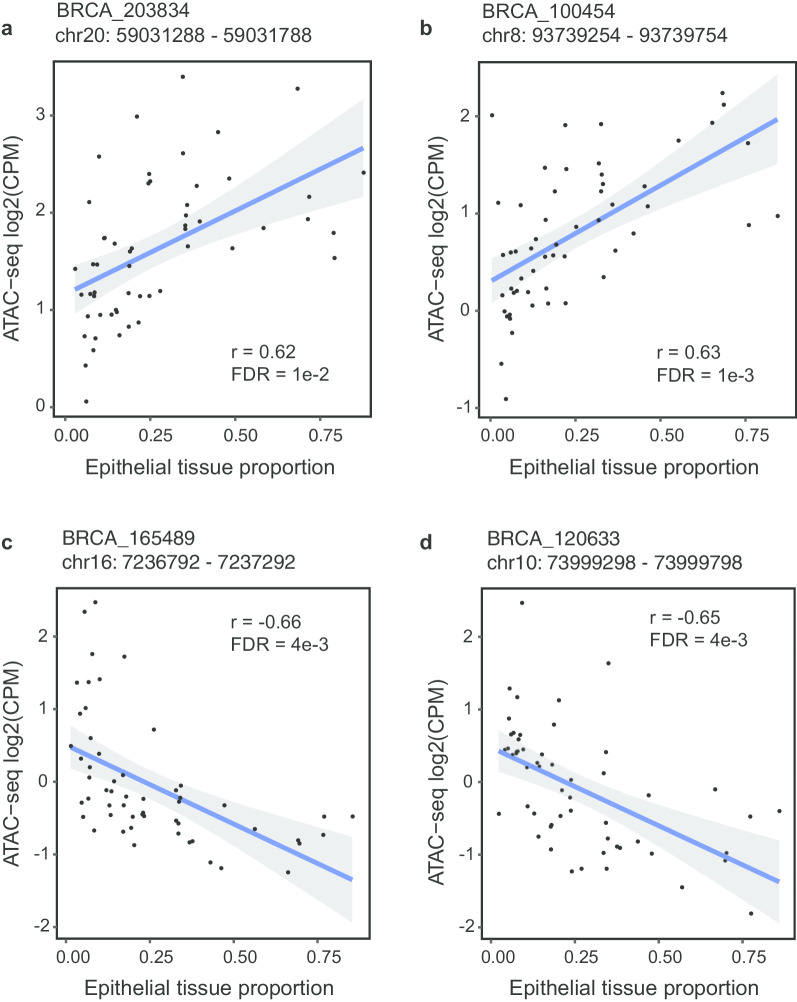
Dot plot of the ATAC-seq accessibility and differential ratio of a peak-to-phenotype link. Each dot represents an individual case. **a** and **b** show examples of a significant positive correlation between ATAC-seq signals and epithelial area proportion. **c** and **d** show examples of a significant negative correlation between ATAC-seq signals and epithelial area proportion

Examples of the correlation between chromatin accessibility and spatial quantification of epithelial tissues are presented in Fig. 3. For the peaks BRCA_203834 and BRCA_100454, the correlation analysis detected a significant positive correlation between the quantitative chromatin accessibility and the epithelial tissue proportion (Fig. 3a-b). A positive correlation indicates that larger epithelial tissue areas appear to have more accessible open chromatin regions. This finding suggests that the regulatory elements in such regions could potentially enhance tumor tissue development. On the contrary, a clear negative correlation between the quantitative chromatin accessibility and the epithelial tissue proportion was observed for peaks BRCA_165489 and BRCA_120633 (Fig. 3c-d). A negative correlation suggests that accessible chromatin regions that are associated with smaller epithelial tissue areas in the tumor might be repressed by regulatory elements in these regions. Taken together, these results demonstrate that this correlation analysis can identify epithelial tissue proportion-associated regulatory regions from ATAC-seq data, which could potentially implicate regulatory elements responsible for cancer development.

### Linking DNA regulatory elements to target genes

We next asked whether the epithelial tissue proportion-associated regulatory regions identified by the correlation analysis could be related to elements of the breast cancer pathway. To address this question, we first identified candidate target genes for the regulatory regions that significantly correlated with the epithelial tissue proportion (see Methods). For putative promoter regions, the closest gene to each region was considered as the target gene. However, because enhancer regions can be far away from their target genes, we used the predicted distal peak-to-gene links obtained from TCGA [16].

In total, 77 regulatory regions were in promoter regions, which we considered as potential promoter regulatory elements, while another 21 regulatory regions were detected by distal peak-to-gene links (< 500 kbp), which we considered potential enhancer regulatory elements. Many of these peak-to-gene links occurred in clusters that were predicted to be linked to the same gene, resulting in a total of 74 target genes that were selected for further downstream analysis. A complete list of these peak-to-gene links and target genes can be found in Additional file 2: Table S4. Since the distal peak-to-gene links were based on the correlation of ATAC-seq accessibility and gene expression across all samples (see Methods), the target genes can be further divided into two groups according to whether the expression data were positively or negatively correlated with the epithelial tissue proportion. Among the 74 target genes, 22 of them were positively correlated with the epithelial tissue proportion while the other 52 genes were negatively correlated. Some important breast cancer and tumor oncogenes, such as *PARI*, *CCNE2* and *RAD54B*, were detected to have positive correlations with epithelial tissue proportion in our study. Previous studies have demonstrated that *PARI* overexpression was correlated with aggressive tumor cell proliferation and poor prognosis in breast cancer [24], high expression of *CCNE2* in breast cancer is strongly predictive of shorter distant metastasis-free survival following endocrine therapy [25] and *RAD54B* potentiates tumor growth and predicts poor prognosis of breast cancer patients [26].

**Fig. 4:**
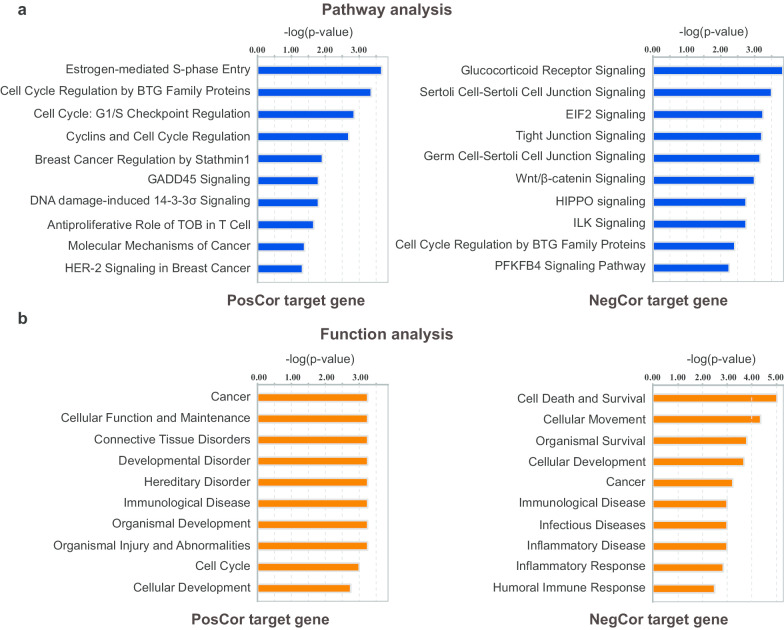
Enrichment of different canonical pathways and functions in genes with regulatory regions that were significantly correlated with epithelial tissue proportions. **a** Canonical pathway analysis results of image feature-positively (PosCor) and image feature-negatively (NegCor) correlated genes. **b** Diseases & Functions analysis results of image feature-positively (PosCor) and image feature-negatively (NegCor) correlated genes. Significance values are presented in –log (*p*-value)

For these 74 target genes, we performed pathway and function enrichment analysis using Ingenuity Pathway Analysis (IPA). We further observed that these genes were enriched in breast cancer-related pathways and functions, especially genes that were positively correlated with epithelial tissue proportion (Fig. 4). For instance, the breast cancer-crucial pathways, *Estrogen-mediated S-phase Entry* and *Breast Cancer Regulation by Stathmin1*, were significant findings from our integrative analysis. In addition, some tissue development and disorder associated functions were specifically enriched, such as *Connective Tissue Disorders* and *Developmental Disorder*. Altogether, these results underscore the ability to utilize our integrative analysis of image and chromatin accessibility data to identify genes that play a role in breast cancer development.

### Integrative analysis enhances the prognostic prediction power

**Fig. 5:**
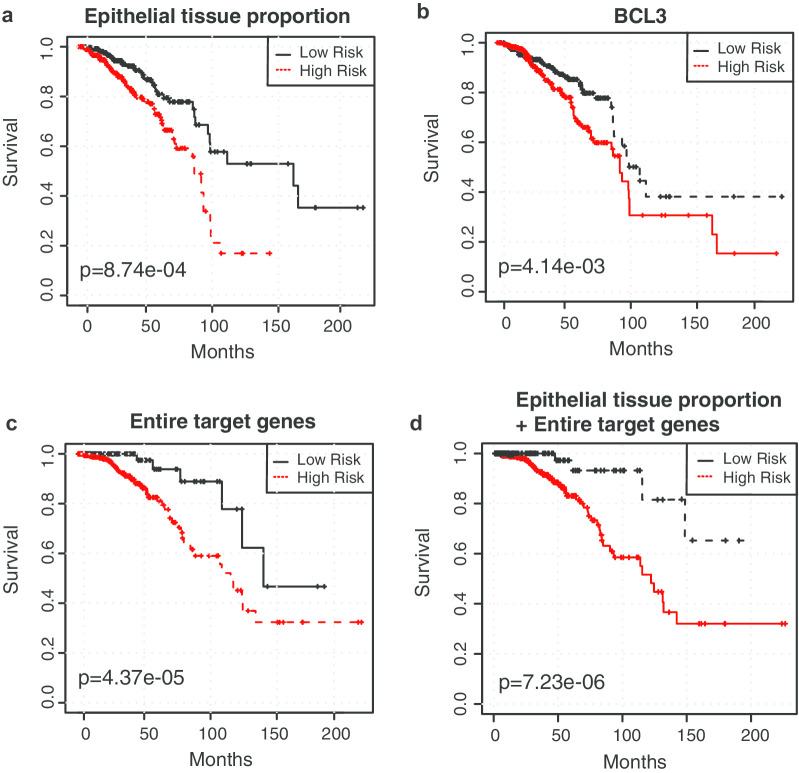
Omics and image features predict the survival outcomes of ER-positive BRCA patients. **a** Univariate survival curve using the epithelial tissue proportion. **b** Univariate survival curve using the expression of one of the identified target genes (*BCL3*). **c** Multivariate survival curve using all of the significant target genes. **d** Multivariate survival curve using all of the significant target genes and the image feature. For panels **a** and **b**, patients were stratified into low-risk and high-risk groups with the median value of each independent variable as a threshold. For panels **c** and **d**, patients were aggregated into low and high-risk groups using a k-means clustering algorithm

We next asked whether the integrative analysis could better predict patient prognosis. To address this question, we first examined the performance of patient stratification when using image features or the target genes alone. For the survival analysis, we used additional ER-positive breast cancer cases with paired histopathological images, gene expression data and survival data available from the TCGA-BRCA cohort. Cases with missing expression data or histopathological images were excluded, leaving a selected set of 663 samples. Target gene expression data and clinical information of these 663 TCGA ER-positive breast cancer cases can be found inAdditional file 2: Table S5. Univariate analysis showed that the epithelial tissue proportion was significantly related to prognosis (Fig. 5a), as were 37.8% (28/74) of the target genes (*p*-value < 0.05). The log-rank test results of all survival-related variables are listed in Additional file 2: Table S6. These results showed that many individual target genes derived from the integrative analysis stratified patients with distinct prognosis. For example, cases with high expression of *BCL3* had significantly worse overall survival than those with low *BCL3* expression (*p* = 0.004, Fig. 5b). Previous studies have proven *BCL3* as an independent prognostic factor [27]. Based on this finding, we performed a multivariate survival analysis using all of the significant univariate features to further investigate whether the integrative analysis would provide better prognostic prediction. As shown in Figs. 5c & d, the integrated multi-modal feature achieved superior stratification performance compared to using the image- or omics-features alone (p_genes + image_ = 7.23e-06, p_genes_ = 4.37e-05, p_image_ = 8.74e-04, p_single gene_ = 0.004). It is noteworthy that the patients with relatively low epithelial tissue proportions showed longer survival (Fig. 5a), which likely reflects the fact that most breast cancers are epithelial tissues and a lower epithelial tissue proportion corresponds to a smaller area of cancer cells. Taken together, these results suggest that the prognostic model based on the target genes and epithelial tissue proportions identified using our integrative framework can be used to effectively guide the risk stratification of ER-positive breast cancer.

## Discussion

The integration of biomedical images with different kinds of omics data has the potential to identify new biomarkers and improve mechanistic understanding of diseases. Nevertheless, screening for the true image feature-associated genomic regulatory regions remains a challenging problem. In this study, we introduced an integrative analysis framework, based on ATAC-seq data and matched histopathological whole-slide images, for detecting gene regulatory regions that correlated with the proportion of epithelial tissue in ER-positive breast cancer.

The major conclusion of this study is that by integrating histopathological images with ATAC-seq data, we can efficiently evaluate associations between chromatin accessibility changes and the epithelial tissue proportion. This conclusion is based on the following evidence: First, we detected epithelial tissue proportion-associated open chromatin regions using canonical correlation analysis. Second, we provided evidence that the target genes of these detected regulatory regions tended to be enriched for breast cancer-related pathways. Interestingly, we found that 40.5% (30/74) of the target genes identified in this study were also identified in our previous work, which directly analyzed the relationship between the epithelial tissue proportion and gene expression data [23]. Importantly, 25 of these 30 genes have been described as breast cancer-associated genes in the literature. For example, independent breast cancer studies have shown that AKT1 suppresses migration and metastasis [28–30] and BCL3 inhibits apoptosis and tumor progression [31]. The enrichment results from our integrative study further support the evidence of how these identified genes contribute to the epithelial tissue proportion. Although some genes, such as *DHX34* and *RELB*, have not yet been shown to be directly related to breast cancer, it’s possible that they could lead to the discovery of new breast cancer related genes or biomarkers. Finally, we found that the integration of identified genes with the epithelial tissue proportion can better stratify patient prognosis compared to either alone. Collectively, these findings demonstrate that the integrative analysis approach presented here can be used to identify potential epithelial tissue proportion-associated regulatory regions, and thereby further our understanding of the molecular mechanisms of complex diseases.

While the integrated analysis approach used in this study revealed a relationship between image morphological and genomic features, there are limitations to this study. First because obtaining matched ATAC-seq and image datasets is challenging, only 54 ER-positive breast cancer samples were used in the correlation analysis. As a result, pairwise correlations between specific chromatin accessibility changes and the image-based epithelial tissue proportion could not be validated in other datasets, which could introduce dataset-specific bias such that the detected open chromatin regions may not represent all regulatory regions in this tumor type. A second limitation is that only the epithelial tissue proportion was used as an image feature in this study. The tumor microenvironment is a complex system and histopathological images demonstrate a complicated distribution of different tissues and cell types. Other histopathological image features have proven to be important for the diagnosis and prognosis of breast cancer, such as the textural features of epithelial tissue [32, 33] and the spatial relationship between tumor cells and tumor-infiltrating lymphocytes [34, 35]. Extracting such features requires a more elaborate image processing system which could identify not only tissues but also different cells. Despite these limitations, our findings should apply more generally to other ER-positive breast cancer cases because of the relatively stringent correlation coefficient (*r* > 0.5) and the multiple testing correction (FDR < 0.5) that were used to minimize false positive findings. Furthermore, the distribution of the epithelial tissues in the 54 cases used in this study were consistent with those in the entire TCGA ER-positive breast cancer cohort, which further supports the generalizability of our findings.

Our future studies will extend this framework to incorporate additional representative image features to further investigate the intrinsic relationship between genotypes and clinical phenotypes. Future comparative ATAC-seq experiments with normal samples are needed to verify our findings. Additionally, to further understand the genetic basis of this disease, future studies could integrate our algorithm with rare variant or eQTL analysis.

## Conclusions

Our analysis demonstrates the ability to integrate chromatin accessibility signals and histological images for exploring the drivers and molecular mechanisms of ER-positive breast cancer. This integrated analysis will enable efficient prioritization of gene regulatory regions identified by correlation studies for further studies to validate their causal regulatory function. Ultimately, identifying regulatory regions and their target genes will further our understanding of the underlying molecular mechanisms of breast cancer. Furthermore, the entire pipeline can be easily applied to different diseases.

## Methods

### Datasets

Chromatin accessibility data, gene expression data, H&E-stained whole-slide histopathology images and matched clinical information were obtained from TCGA and can be downloaded from the link provided in the Data Availability section. Both the ATAC-seq and mRNA expression data were obtained from frozen tissue sections in proximity to the sections that were used to generate the H&E-stained tissue slides [36]. The 74 BRCA samples with quantitative chromatin accessibility data were obtained from TCGA ATAC-seq cohort. Among these, 58 samples were categorized as ER-positive, based on the clinical annotation data. Matched histopathological images and gene expression data for 1000 breast cancer cases were obtained from TCGA BRCA cohort. Among these, 663 samples were categorized as ER-positive and were chosen for the survival analysis. Four samples from the ATAC-seq cohort with missing image data were excluded. The remaining 54 ER-positive breast cancer samples were chosen for correlation analysis. Demographic and clinical characteristics of the selected cases used in this study are listed in Table 1.

**Table 1:** Demographic and clinical characteristics for TCGA breast cancer patients

Cohort		TCGA BRCA	
Analysis type		Correlation analysis	Survival analysis
Total cases (No.)		74	1092
ER-positive cases (No.)		58	773
Age (years)	Range	34 ~ 80	26 ~ 90
	Median	58	60
Follow-up (days)	Range	348 ~ 4275	1 ~ 7067
	Median	956	1313
Data category	ATAC-seq	58	N/A
	Image (tissue slide)	54	773
	RNA-seq	N/A	663
	Matched cases	54 (ATAC-seq and image)	663 (RNA-seq and image)

The TCGA BRCA cohort provides two types of H&E stained whole-slide images: tissue slides and diagnostic slides. Tissue slides are sections from frozen tumor specimens that are typically used to determine whether the tumor borders are clean. Diagnostic slides are formalin-fixed paraffin-embedded (FFPE) sections, which typically have better preservation of cell morphology; however, these sections frequently show areas with tissue damage. Only tissue slides were used for the histopathological images in this study. Histopathological images were downloaded in the native image format as Aperio SVS files. Each image was acquired at a 40X objective lens using Aperio Scanscope, with each pixel corresponding to a 0.24 × 0.24 square micron area.

### Algorithm for tissue quantification on tissue slides

We used our previously described whole-slide image-processing framework [23] to calculate the epithelial tissue proportion from histopathological images. This framework first employed a convolutional neural network (CNN) segmentation model to classify the epithelial and stromal tissues. The CNN model was trained on an independent image cohort and validated on TCGA tissue slides. Then based on the tissue segmentation results derived from CNN model, we calculated the epithelial tissue proportion as:

$${Proportion}_{epi}={Area}_{epi}/\left({Area}_{epi}+{Area}_{stro}\right)$$

where *Proportion*_*epi*_ represents the epithelial tissue proportion and *Area*_*epi*_ and *Area*_*stro*_ represent the epithelial and stromal tissue area identified by the CNN model, respectively.

### Omics-image correlation analysis

Associations between chromatin accessibility changes and the epithelial tissue proportion were determined using canonical correlation analysis. Specifically, for each detected open chromatin region, chromatin accessibility was quantified using the normalized count from that specific region for each case. The Spearman correlation coefficients *r* between each quantitative chromatin accessibility and epithelial tissue proportion were calculated across all ER-positive BRCA cases. Given the correlation coefficient *r* and the sample size, the *P*-value for the correlation coefficient was calculated using the exact permutation distributions for the two-tailed test. The FDR was calculated following the Benjamini–Hochberg procedure [37]. Finally, open chromatin regions were considered significantly correlated with epithelial tissue proportions when FDR < 0.05.

### Linking DNA regulatory elements to genes

To associate regulatory regions with the genes they are predicted to regulate, we adopted the same procedure as TCGA [16]. Specifically, a promoter region was defined to lie within 1000 to 100 bp upstream of transcription start site (TSS). The promoter peak-to-gene mapping information was derived from peak summits located within the promoter region of a gene.

The distal peak-to-gene link was based on the correlation of ATAC-seq accessibility and gene expression across all samples. All peaks whose summit were located within 500 kbp from a gene’s TSS were considered. A conservative FDR cutoff of 0.01 was used to avoid false positives. Putative enhancer peaks were further filtered if (i) the correlation with gene expression was strongly driven by DNA copy number amplification, or (ii) links involved an ATAC-seq peak that overlapped the promoter of any gene.

### Canonical pathway and function enrichment analysis

Ingenuity Pathways Analysis (IPA, Qiagen) was used to explore possible signaling pathways and functions for genes whose regulatory region was correlated with epithelial tissue proportion. IPA core analyses was conducted for each identified gene using experimentally observed knowledge in the Ingenuity Knowledge Base. Pathway analysis was conducted using *Canonical Pathways* and function analysis was derived from *Diseases & Functions.*

### Machine-learning methods for prognostic prediction

For the univariate survival analysis, we used the median value of each feature to stratify cases into low-risk and high-risk groups. The Kaplan-Meier method and log-rank test were used to fit the survival data and test for survival difference between the two groups.

For the multivariate survival analysis, a previously described method utilizing a k-means clustering algorithm [38] was implemented to aggregate the patients into low-risk and high-risk groups, before testing if these 2 subgroups had distinct survival outcomes using the log-rank test.

## Supplementary Information

**Additional file 1: Supplemental Figure 1**. Representative H&E stained histopathology tissue image of TCGA breast cancer cases. A) Original H&E stained histopathology image and paired tissue segmentation result of a high-epithelium case. B) Original H&E stained histopathology image and paired tissue segmentation result of a low-epithelium case. The tissue segmentation results were derived from our previous work, with the red, green and black regions corresponding to epithelial and stromal tissue and background in the original image, respectively.

**Additional file 2: Supplemental Table 1**. The epithelial tissue proportion data of 54 TCGA ER-positive BRCA cases. **Supplemental Table 2**. The ATAC-seq peak signal data of 54 TCGA ER-positive BRCA cases. **Supplemental Table 3**. Significant epithelial tissue proportion-associated peaks and their target genes. **Supplemental Table 4**. Promoter and distal enhancer peak-to-gene links and their target genes. **Supplemental Table 5**. Expression data of the identified genes and clinical information for 663 TCGA ER-positive BRCA cases. **Supplemental Table 6.** Results of the univariate survival analysis.

## Data Availability

All raw and processed data are freely available from TCGA, BRCA ATAC-seq hub (https://atacseq.xenahubs.net) and BRCA Genomic Data Commons (GDC) hub (https://gdc.cancer.gov). Promoter peak location information was obtained from the file (https://atacseq.xenahubs.net/download/brca/brca_peak_Log2Counts_dedup_promoter). Enhancer peak location information was obtained from the file (https://atacseq.xenahubs.net/download/brca/brca_peak_Log2Counts_dedup_hQlinkage).

## Abbreviations

ATAC-seq: Assay for transposase-accessible chromatin using sequencing
TCGA: The Cancer Genome Atlas
CNN: Convolutional neural network
BRCA: Breast cancer
TSS: Transcription start site
H&E: Hemotoxin and eosin
