# Supplementary material for: Integrative analysis of histopathological images and chromatin accessibility data for estrogen receptor-positive breast cancer
Source: BMC Med Genomics. 2020 Dec 28;13(Suppl 11):195. doi: 10.1186/s12920-020-00828-4 (PMC7771206; doi:10.1186/s12920-020-00828-4)
Supplement: Supplementary file 1 — Additional file 1: Supplemental Figure 1. Representative H&E stained histopathology tissue image of TCGA breast cancer cases. A) Original H&E stained histopathology image and paired tissue segmentation result of a high-epithelium case. B) Original H&E stained histopathology image and paired tissue segmentation result of a low-epithelium case. The tissue segmentation results were derived from our previous work, with the red, green and black regions corresponding to epithelial and stromal tissue and background in the original image, respectively. [file 12920_2020_828_MOESM1_ESM.pdf]

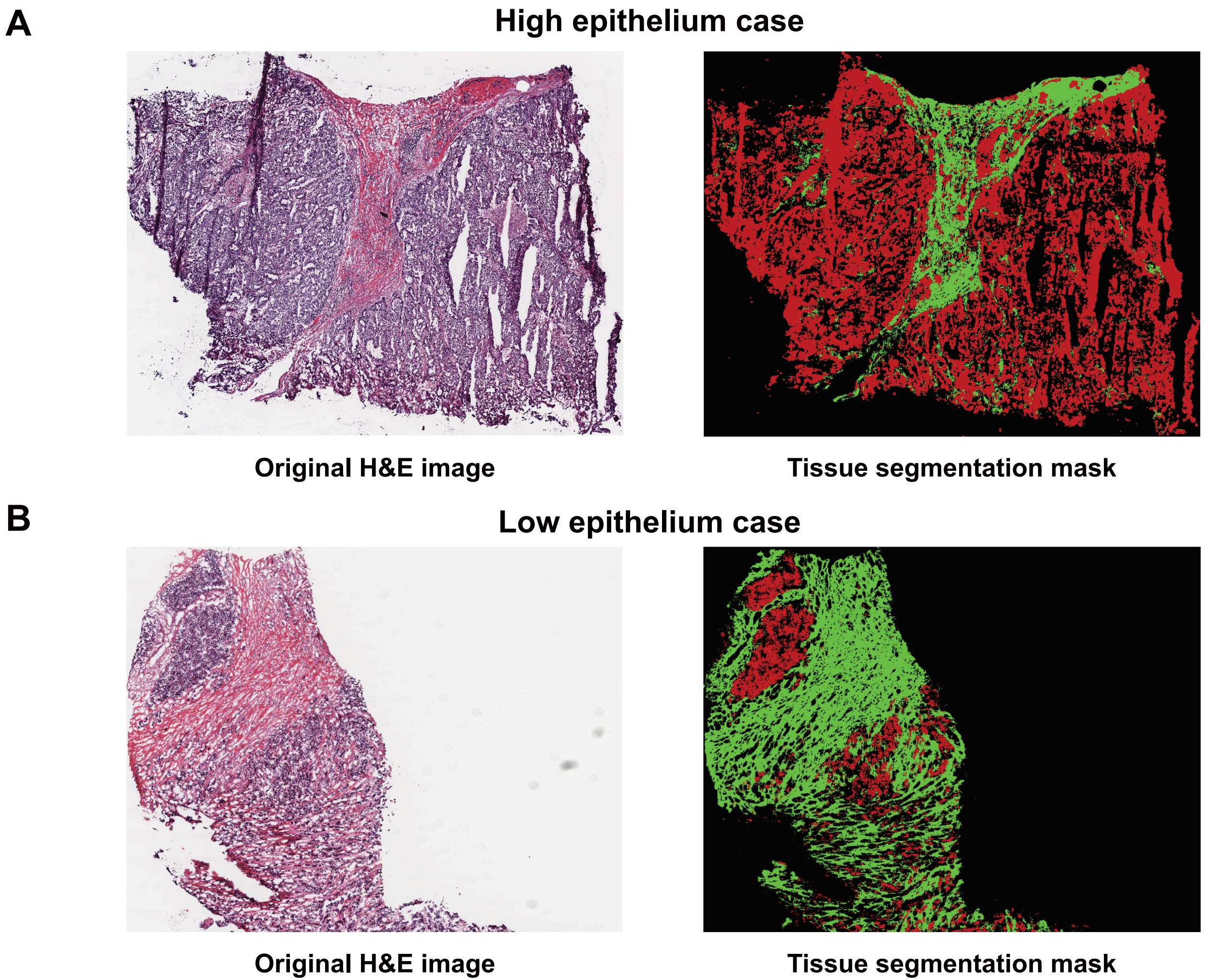
A
High epithelium case
Original H&E image
Tissue segmentation mask
B
Low epithelium case
Original H&E image
Tissue segmentation mask
